# Pitfalls in PSMA imaging: [^18^F]rhPSMA-7-PET/CT reveals presence of chrondrosarcoma

**DOI:** 10.1007/s00259-021-05254-x

**Published:** 2021-02-25

**Authors:** B. Feuerecker, C. Mogler, K. Wörtler, C. Knebel, M. Eiber, M. Krönke

**Affiliations:** 1 School of Medicine, Department of Nuclear Medicine, Klinikum rechts der Isar, Technical University of Munich, Munich, Germany; 2 Deutsches Konsortium für translationale Krebsforschung (DKTK), Heidelberg, partnersite München and German Cancer Research Center (DKFZ), Heidelberg, Germany; 3 School of Medicine, Department of Radiology, Klinikum rechts der Isar, Technical University of Munich, Munich, Germany; 4 School of Medicine, Department of Pathology, Klinikum rechts der Isar, Technical University of Munich, Munich, Germany; 5 School of Medicine, Department of Orthopedic Surgery, Klinikum rechts der Isar, Technical University of Munich, Munich, Germany

PET imaging targeting the prostate membrane-specific antigen (PSMA) has demonstrated high potential in localizing disease in prostate cancer. In addition to prostate cancer, both benign and malignant lesions have been reported to highly express PSMA (e.g. ganglia, parathyroid adenoma, lung, thyroid, and renal cancers [1–3]). Here, we describe the case of a 71-year-old male patient who was referred for imaging of biochemical recurrent prostate cancer (Gleason Score 7b, iPSA 116 ng/ml, s/p radical prostatectomy, PSA at imaging 0.32 ng/ml). [^18^F]rhPSMA-7-PET/CT showed high PSMA-ligand uptake in an expansile lytic bone lesion of the sternum, a small local recurrence and a bone lesion. The lesion of the sternum showed a lobular appearance, a shell-like periosteal reaction, and matrix calcifications (A PET MIP, B fused PET/CT, C PET only, D axial CT only, E sagittal CT only showing a sharply demarcated lesion, red arrows indicate the lesion, green arrow indicates a bone lesion, pink a local recurrence) and exhibited high T2-weighted signal intensity (F) and septonodular contrast enhancement (G) on magnetic resonance imaging. Given this morphological appearance, metastasis seemed unlikely and the subsequent biopsy and surgery confirmed the diagnosis of a grade 3 chondrosarcoma. Immunohistochemistry showed that PSMA was mainly expressed in small intralesional blood vessels/capillaries (histological slices H, yellow arrow: PSMA-staining; blue arrow: hyaline matrix). This is in line with previous reports demonstrating high PSMA-expression in vascular regions [2, 4, 5].

In summary, this case illustrates the potential pitfalls in PSMA-ligand PET-imaging given that PSMA-expression is also present in non-prostate cancer lesions and highlights the necessity of thorough analysis of the accompanying cross-sectional imaging modality.


